# Substances use between early and later stages of the COVID-19 pandemic in Israel

**DOI:** 10.1186/s13584-021-00484-8

**Published:** 2021-08-12

**Authors:** Hagit Bonny-Noach, Keren Cohen-Louck, Inna Levy

**Affiliations:** 1 Department of Criminology, Ariel University, Ariel, Israel; 2 Department of Interdisciplinary Studies, Zefat Academic College, Safed, Israel

**Keywords:** COVID-19, Substance use, Cannabis, Alcohol, Israel

## Abstract

**Aims:**

Only several empirical studies have examined substance use during the COVID-19 pandemic in general populations. Most of these studies compared self-reported substances use before the pandemic and during the pandemic's early stages. This study aims to identify the changes in substance use between the early and later waves of the COVID-19 pandemic in Israel.

**Methods:**

A cross-sectional online survey recruited 750 participants (ages 18–65) in two waves: (1) 427 during and following the first lockdown (April-mid-May, 2020); and (2) 323 following the second lockdown (from October to mid-November, 2020).

**Results:**

Participants who experienced two lockdowns reported more frequent consumption of all alcoholic beverages and cannabis in the last 30 days than those who experienced one lockdown. After controlling for demographic variables, significant differences were found between participants who experienced one lockdown and those who experienced two lockdowns in the consumption of alcoholic beverages (*F*(1, 742) = 6.90, *p* = .01, *η*^2^ = .01). However, there was no significant association between pandemic duration and other illegal drug consumption.

**Conclusions:**

There is a significant association between pandemic duration and alcohol consumption. Policymakers and practitioners should develop national alcohol and cannabis use prevention and harm reduction interventions during pandemics with a focus on men, singles and youth.

## Introduction

The COVID-19 pandemic outbreak has caused mass trauma [1] and victimization [2], posing an unprecedented threat to global wellbeing and affecting the daily lives of individuals worldwide. During the COVID-19 pandemic, many people have experienced strong fears [2–4], anxiety and depression [5–8]. Stressors caused by this challenging period can lead to increased substance use [9], but COVID-19’s impact on drug demand and supply is unknown and hard to predict. However, it could be far-reaching [10].

Only a few empirical studies examined COVID-19's impact on substance consumption in the general population [9, 11]. Most of those studies were conducted in the early stages of COVID-19 and included questions about substance use behaviors before and during the pandemic. Their findings indicate that alcohol [12–14], tobacco [13], and cannabis [11] consumption increased during the COVID-19 pandemic compared to the period before COVID-19. In addition to increased consumption, some individuals started consuming new substances, including marijuana and psychopharmaceuticals [15]. The increase in consumption was associated with COVID-19 related fears, and substance use was meant to cope with these fears [16, 17]. Additional drug use tendencies have been further excaserbated during the COVID-19 pandemic due to increased difficulties in providing prevention, harm-reduction and treatment services [19, 20].

In Israel, only a few studies examined substance use in the pandemic period. These studies indicate that during the early stage of COVID-19, there were increased demands for admission to detoxification centers and addiction treatment services [19], increased craving for drugs, alcohol, and tobacco consumption among individuals recovering from substance abuse [21], and increased consumption of substances among social-work students [8]. The only study that compared the early and later stages of the pandemic in a sample of social-work students found no significant differences based on pandemic duration [22]. Thus, to the best of our knowledge, there is no data on substance use patterns during the COVID-19 pandemic in the general population in Israel, and data on the effects of pandemic duration is limited. The current research aims to determine the patterns of substance consumption in Israel's general population, compare the early and later stages of the pandemic, and identify the association between substance consumption and participants' demographic characteristics.

## Method

### Procedure

Following Ariel University's ethics committee approval, we conducted an online survey via social media outlets (e.g., Facebook, WhatsApp). The survey was distributed as a snowball sample among co-workers, students, and their families and friends. We distributed the questionnaire following the first lockdown (April-mid-May, 2020) and following the second lockdown (October-mid-November, 2020). The opening statement clarified that the questionnaire was anonymous, and participants could stop answering the questionnaire at any stage. At the end of the survey, we included information on crisis and call centers for mental help.

### Participants

The survey included 750 participants, ages 18–65 (*Mean* = 29.79, *S.D*. = 11.81). The majority were female (72.8%), single (67.7%), secular (50.8%), and academically educated (75.5%). Over half (56.9%) of the respondents participated in this study during the early stage of the COVID-19 pandemic in Israel (March to mid-May, 2020), following the first lockdown and the rest following the second lockdown (October to mid-November, 2020). The gender distribution was similar in both samples. Compared to the first wave sample, the second wave sample was characterized by a higher frequency of singles and lower education and economic status (Table 1).

**Table 1:** Socio-demographic characteristics in total sample and by lockdowns

Demographics	COVID-19 lockdowns		Total *N* = 750 (%)	*χ*^*2*^	*df*	*Cramer’s v*
	One lockdown (*n* = 427) (%)	Two lockdowns *(n* = 323) (%)				
*Gender*						
Female	73.5	71.8	72.8	.27	1	.02
Male	26.5	28.2	27.2			
*Family status*						
Single	60.2	77.6	67.7	25.60***	2	.19***
Married	36.2	19.6	29.0			
Divorced	3.6	2.8	3.3			
*Educational level*						
High school	19.9	32.8	25.5	16.15***	1	− .15
Academic	80.1	67.2	75.5			
*Economic status*						
Low	55.8	75.9	70.1	20.15***	2	.21***
Average	19.4	13.6	15.3			
High	24.8	10.5	14.6			
*Religiosity*						
Secular	55.8	48.8	50.8	2.93	2	.08
Traditional	20.2	27.6	25.5			
Religious	24.0	23.6	23.7			

***p < .001

### Measures

*Demographic variables*: The questionnaire gathered information on age, gender, family status, religiosity, education, employment, and economic status.

*Alcohol and drug consumption*: To examine substance consumption, we used a questionnaire adapted from Israel's National Epidemiological Survey [23]. The participants ranked how many times during the last 30 days they used the following substances: wine (excluding religious rituals and practices); beer; hard liquor/spirits; cannabis; other illegal substances (e.g., MDMA, cocaine, LSD). The ranking options varied from 0 (never) to 6 (30 + times). We also assessed binge drinking, heavy episodic use of consuming five or more alcoholic beverages in the span of a few hours, on the same ranking scale. The overall measure of alcohol and drug consumption was the mean score for alcohol and the mean score for drugs.

*Pandemic duration*: To examine the effect of pandemic duration we compared the participants who experienced one lockdown to a separate group of participants who experienced two lockdowns.

### Data analysis

Analyses were carried out using SPSS Version 25. We used chi-square to assess psychoactive substance consumption frequency and the association between substance consumption and research variables. The nature of the association of demographic variables and substance consumption did not differ by pandemic duration (1st vs. 2nd wave). Therefore, we present the results regarding the whole sample. We used ANCOVA to examine the effects of gender and pandemic duration while controlling for the factors, which were associated with the type of sample: family status, income and educational level. At first, we examined the association between demographic characteristics and substance consumption, and then conducted ANCOVA to examine the effects of the pandemic duration regarding the overall measures while controlling for demographic factors (age, education, income) associated with the type of sample (1st wave/2nd wave).

## Results

### Pandemic duration

**Fig. 1:**
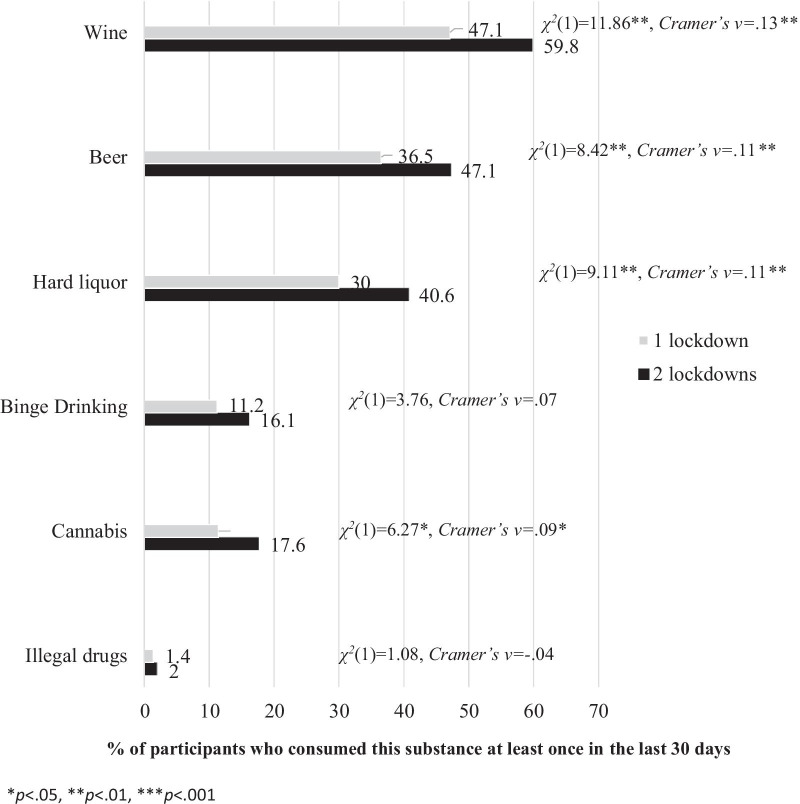
Graphic comparison in frequency of consumption between the participants who experienced one lockdown and those who experienced two lockdowns by type of substance

There is a significant association between substance consumption and pandemic duration in most consumption aspects. Figure 1 indicates that there is a significant association between the pandemic duration and consumption of wine, beer, hard liquor, and cannabis. The participant who experienced two lockdowns reported higher frequencies of wine, beer, hard liquor, and cannabis consumption than the participants who experienced only one lockdown.

To examine the general effect of the pandemic duration, we conducted an ANCOVA and controlled for age, income (lower than average: 1 = lower, 0 = all else; higher than average income: 1 = higher, 0 = all else), and education (0 = high school level, 1 = academic level). Considering the strong correlation between age and unmarried family status (*r* = -0.74, *p* < 0.001), we did not control for family status. Table 2 shows that there are significant main effects of gender (*F*(1, 742) = 31.58, *p* = 0.00, *η*^*2*^ = 0.04) and lockdown (*F*(1, 742) = 6.90, *p* = 0.01, *η*^*2*^ = 0.01) regarding the overall alcohol measure. In general, men reported higher alcohol consumption than women. As for the pandemic duration, participants who experienced two lockdowns reported higher alcohol consumption than participants who experienced one lockdown. Regarding binge drinking, there was a significant effect of pandemic duration (*F*(1, 742) = 4.81, *p* = 0.03, *η*^*2*^ = 0.01), but no significant effect of gender (*F*(1, 742) = 2.20, *p* = 0.14, *η*^*2*^ = 0.00). Participants who experienced two lockdowns reported significantly higher binge drinking than participants who experienced only one lockdown. As for the overall measure of drug consumption there were no significant effects of gender (*F*(1, 742) = 3.39, *p* = 0.07, *η*^*2*^ = 0.01) and pandemic duration (*F*(1, 742) = 0.46, *p* = 0.49, *η*^*2*^ = 0.01).

**Table 2:** Differences in overall alcohol and drug consumption and in binge drinking by gender and pandemic duration: means and standard errors

	Overall measure of alcohol consumption Mean (S.E.)	Binge drinking Mean (S.E.)	Overall measure of drug consumption Mean (S.E.)
*Gender*			
Female	1.67 (.04)	0.22 (.03)	1.21 (.03)
Male	2.09 (.06)	0.31 (.05)	1.32 (.05)
*Pandemic duration*			
One lockdown	1.76 (.05)	0.19 (.04)	1.24 (.04)
Two lockdowns	2.00 (.06)	0.34 (.05)	1.29 (.05)

### Demographic characteristics and substances consumption

The association between gender and substance consumption was significant only regarding beer and hard liquor (Table 3). More men consumed beer and hard liquor than women. There was no significant association between gender and consumption of wine, cannabis, other illegal drugs and no significant association was found between gender and binge drinking.

**Table 3:** Association between gender and alcohol and drug consumption

Consumption	Gender		*χ*^*2*^ *(df* = *1)*	*Cramer's v*
	Women *(n* = 546) %	Men *(n* = 204) %		
*Wine*				
No	48.7	44.1	1.26	.04
Yes	51.3	55.9		
Total (%)	100	100		
*Beer*				
No	66.8	37.7	51.98***	.26
Yes	33.2	62.3		
Total (%)	100	100		
*Hard liquor*				
No	68.9	56.4	10.25**	.18
Yes	31.1	43.6		
Total (%)	100	100		
*Binge drinking*				
No	87.9	83.3	2.69	.06
Yes	12.1	16.7		
Total (%)	100	100		
*Cannabis*				
No	87.2	82.8	2.32	.06
Yes	12.8	17.2		
Total (%)	100	100		
*Other illegal drugs*				
No	99.3	98.0	2.12	.05
Yes	0.7	2.0		
Total (%)	100	100		

**p < .01, ***p < .001

Table 4 shows that the association between consumption and family status was statistically significant regarding wine, beer, hard liquor, binge drinking, and cannabis. Among participants who were single, there was a higher frequency of wine, beer, hard liquor, and cannabis consumption, at least once during the last 30 days than among married and divorced participants. Additionally, for singles, binge drinking in the last 30 days was more frequent than among married and divorced participants. There was no significant association between family status and the consumption of other illegal drugs.

**Table 4:** Association between family status and alcohol and drug consumption

Consumption	Family status			*χ*^*2*^ *(df* = *1)*	*Cramer's v*
	Single *(n* = 502) %	Married *(n* = 215) %	Divorced *(n* = 24) %		
*Wine*					
No	44.4	53.5	62.5	7.16*	.10
Yes	55.6	46.5	37.5		
Total (%)	100	100	100		
*Beer*					
No	56.8	60.9	83.3	7.22*	.10
Yes	43.2	39.1	16.7		
Total (%)	100	100	100		
*Hard liquor*					
No	59.8	77.7	79.2	23.43***	.18
Yes)	40.2	22.3	20.8		
Total (%)	100	100	100		
*Binge drinking*					
No	83.1	93.0	100	16.65***	.15
Yes	16.9	7.0	0		
Total (%)	100	100	100		
*Cannabis*					
No	81.7	95.3	95.8	25.49***	.19
Yes	18.3	4.7	4.2		
Total (%)	100	100	100		
*Other illegal drugs*					
No	98.8	99.5	100	1.09	.04
Yes	1.2	0.5	0		
Total (%)	100	100	100		

**p < .01, ***p < .001

There was a significant, negative and weak correlation between age and consumption of beer, hard liquor, cannabis, and binge drinking (Table 5). There was no significant correlation between age and consumption of wine and illegal drugs. There was a significant difference in age by family status (*F*(2, 738) = 503.11, *p* = 00). Single participants were the youngest (*Mean* = 23.72, *S.D.* = 4.49), followed by married (*Mean* = 41.08, *S.D.* = 12.19), and lastly divorced participants (*Mean* = 52.90, *S.D.* = 8.02).

**Table 5:** Association between age, education and income and alcohol and drug consumption (n = 750)

Consumption type	Age	Education		Income	
	*r*	*χ*^*2*^*(df* = *1)*	*Cramer's v*	*χ*^*2*^*(df* = *2)*	*Cramer's v*
Wine	− .04	0.08	− .01	3.97	.09
Beer	− .11**	0.34	.02	.16	.02
Hard liquor	− .14***	0.79	− .03	4.73	.10
Binge Drinking	− .12***	0.76	− .03	3.93	.09
Cannabis	− .17***	0.62	− .03	2.96	.08
Other illegal drugs	− .05	2.56	− .06	4.89	.10

**p < .01, ***p < .001

As Table 5 indicates, there was no significant association between education and consumption of wine, beer, hard liquor, cannabis, and other illegal drugs. Educational level was not significantly associated with binge drinking. Also, there was no significant association between income and wine, beer, hard liquor, cannabis, and illegal drug consumption. Income was not significantly associated with binge drinking.

## Discussion

The current study examined associations between demographic characteristics, pandemic-duration, and substance use during the COVID-19 pandemic in Israel. There was a significant association between cannabis consumption and the pandemic duration, but after controlling for demographic variables, the effect of the pandemic duration on the overall drug consumption in the current study was statistically nonsignificant. However, contrary to Reznik et al.'s [22] findings, our study participants who experienced two lockdowns reported significantly higher consumption of alcohol than participants who experienced only one lockdown. The difference in findings regarding alcohol between our study and that of Reznik et al. [22] may be related to the difference in the sample populations. Reznik et al.'s [22] examined social-work students, and we studied the general population.

It is well known that alcohol and drug consumption are related to continuous stress and anxiety [24, 25]. However, there are several possible explanations for the fact that the COVID-19 duration was associated with alcohol and not with drug consumption. Alcohol in Israel is legal and was available during the COVID-19 lockdowns, while drug consumption in Israel is illegal and is considered less mainstream [26]. Cannabis has been somewhat decriminalized in recent years and is relatively available. However, alcohol availability far exceeds that of cannabis [27, 28]. These findings may be also explained by the data's self-reporting nature. It is possible that people feel less comfortable reporting illegal substance use. This tendency to underreport drug use may be especially salient during the COVID-19 pandemic because Israeli security agencies used their anti-terrorism phone-tracking technology to map infections and surveil infected citizens [14]. Citizens may not have trusted the Israeli security agencies to limit themselves to pandemic-related tracking and may have been afraid that our survey reports will be tracked as well.

Additional intriguing findings address gender. The gender differences were significant only regarding beer and hard-liquor consumption, with men reporting higher levels than women. This pattern is surprising, since gender differences are usually more salient [17, 30, 31]. The absence of gender differences regarding binge drinking and drug consumption may suggest that the impact of COVID-19 is beyond gender or traditional gender roles, and women reported similar patterns of binge drinking and drug consumption as men. In addition, it may be that the participants in the current study did not feel a need to binge or use drugs.

### Limitations and future studies

The study's samples are convenience samples. Therefore, we recommend conducting a comprehensive national survey. Additionally, the external validity of this study may be limited, since this study did not address such variables as prior addiction status and job loss. Due to circumstances rapidly changing during the pandemic, future surveys should examine consumption during the pandemic's last three months and last week. The results may also be affected by social desirability or the fear of reporting illegal drug use.

## Conclusions

This study's key finding emphasizes the significant role of pandemic duration as a tendency for alcohol use. The findings indicate a strong need to establish a national alcohol prevention and harm reduction interventions during viral pandemics. As for specific groups that exhibit higher tendency for alcohol consumption, it appears that future studies and interventions should focus on the association between gender, family status, age and alcohol consumption.

## Data Availability

The authors have the data.

## Abbreviation

COVID-19: Coronavirus disease 2019

## References

[1] Gonçalves PD, Moura HF, da Amaral RA, Castaldelli-Maia JM, Malbergier A (2020). Alcohol use and COVID-19: can we predict the impact of the pandemic on alcohol use based on the previous crises in the 21st century? A brief review. Front Psychiatry.

[2] Cohen-Louck K, Levy I (2020). Viruism: The need for a new term describing COVID-19 impact in context of viral victimization. Psychol Trauma Theory Res Pract Policy.

[3] Shechory Bitton M, Laufer A (2021). Mental health and coping in the shadow of the COVID-19 pandemic: the Israeli case. Front Public Health.

[4] Levy I, Cohen-Louck K (2021). Predicting individual function during COVID-19 lockdown: depression, fear of COVID-19, age, and employment. Front Psychol.

[5] Wang C, Pan R, Wan X, Tan Y, Xu L, Ho CS (2020). Immediate psychological responses and associated factors during the initial stage of the 2019 Coronavirus Disease (COVID-19) epidemic among the general population in China. Int J Environ Res Public Health.

[6] Wang Y, Shi L, Que J, Lu Q, Liu L, Lu Z (2021). The impact of quarantine on mental health status among general population in China during the COVID-19 pandemic. Mol Psychiatry.

[7] Hou F, Bi F, Jiao R, Luo D, Song K (2020). Gender differences of depression and anxiety among social media users during the COVID-19 outbreak in China: a cross-sectional study. BMC Public Health.

[8] Yehudai M, Bender S, Gritsenko V, Konstantinov V, Reznik A, Isralowitz R (2020). Covid-19 fear, mental health, and substance misuse conditions among university social work students in Israel and Russia. Int J Ment Health Addict..

[9] McKay D, Asmundson GJG (2020). Substance use and abuse associated with the behavioral immune system during COVID-19: the special case of healthcare workers and essential workers. Addict Behav.

[10] UNODC. World drug report. United Nations publication; 2020. Report No.: Sales No. E.20.XI.6.

[11] Rolland B, Haesebaert F, Zante E, Benyamina A, Haesebaert J, Franck N (2020). Global changes and factors of increase in caloric/salty food intake, screen use, and substance use during the early COVID-19 containment phase in the general population in France: survey study. JMIR Public Health Surveill.

[12] Lechner WV, Laurene KR, Patel S, Anderson M, Grega C, Kenne DR (2020). Changes in alcohol use as a function of psychological distress and social support following COVID-19 related University closings. Addict Behav.

[13] Sun Y, Li Y, Bao Y, Meng S, Sun Y, Schumann G (2020). Brief report: increased addictive internet and substance use behavior during the COVID-19 pandemic in China. Am J Addict.

[14] Vanderbruggen N, Matthys F, Van Laere S, Zeeuws D, Santermans L, Van den Ameele S (2020). Self-reported alcohol, tobacco, and cannabis use during COVID-19 lockdown measures: results from a web-based survey. Eur Addict Res.

[15] Ruiz P, Semblat F, Pautassi R (2021). The change in psychoactive substance consumption in relation to psychological stress during the Covid-19 pandemic in Uruguay. Sultan Qaboos Univ Med J SQUMJ.

[16] Rehm J, Kilian C, Ferreira-Borges C, Jernigan D, Monteiro M, Parry CDH (2020). Alcohol use in times of the COVID 19: implications for monitoring and policy. Drug Alcohol Rev.

[17] Rodriguez LM, Litt DM, Stewart SH (2020). Drinking to cope with the pandemic: The unique associations of COVID-19-related perceived threat and psychological distress to drinking behaviors in American men and women. Addict Behav.

[18] EMCDDA. Impact of COVID-19 on drug markets, use, harms and drug services in the community and prisons [Internet]. Euripean Monitoring Centre for Drugs and Drug Addiction; 2021 Apr. https://www.emcdda.europa.eu/system/files/publications/13745/TD0321143ENN_002.pdf.

[19] Rosca P, Shapira B, Neumark Y (2020). Isolating the isolated: Implications of COVID-19 quarantine measures on in-patient detoxification treatment for substance use disorders. Int J Drug Policy.

[20] Marsden J, Darke S, Hall W, Hickman M, Holmes J, Humphreys K (2020). Mitigating and learning from the impact of COVID-19 infection on addictive disorders. Addiction.

[21] Bonny-Noach H, Gold D (2020). Addictive behaviors and craving during the COVID-19 pandemic of people who have recovered from substance use disorder. J Addict Dis.

[22] Reznik A, Gritsenko V, Konstantinov V, Yehudai M, Bender S, Shilina I (2021). First and second wave COVID-19 fear impact: Israeli and Russian social work student fear, mental health and substance use. Int J Ment Health Addict.

[23] Ezrachi Y, Harel-Fisch Y (2017). Ha shimush be homarim psichoactivim be kerev ha uhlusia ha bogeret be Israel—Ha seker ha epidemology ha- 8 ha arzi [Psychoactive drug use among the adult population in Israel- 8th National epidemiological survey].

[24] Kaysen D, Dillworth TM, Simpson T, Waldrop A, Larimer ME, Resick PA (2007). Domestic violence and alcohol use: trauma-related symptoms and motives for drinking. Addict Behav.

[25] McFarlane AC (1998). Epidemiological evidence about the relationship between PTSD and alcohol abuse: the nature of the association. Addict Behav.

[26] Aviad-Wilchek Y, Levy I, Ben-David S (2017). Readiness to use psychoactive substances among second-generation adolescent immigrants and perceptions of parental immigration-related trauma. Subst Use Misuse.

[27] MacMillan T, Corrigan MJ, Coffey K, Tronnier CD, Wang D, Krase K (2021). Exploring factors associated with alcohol and/or substance use during the COVID-19 pandemic. Int J Ment Health Addict.

[28] Wardell JD, Kempe T, Rapinda KK, Single A, Bilevicius E, Frohlich JR (2020). Drinking to cope during COVID-19 pandemic: the role of external and internal factors in coping motive pathways to alcohol use, solitary drinking, and alcohol problems. Alcohol Clin Exp Res.

[29] Lieber D. Israel turns to its spy agencies to combat coronavirus. Wall Street J. 2020. https://www.wsj.com/articles/israel-turns-to-its-spy-agencies-to-combat-coronavirus-11584735025.

[30] Bonny-Noach H, Shechory-Bitton M (2020). Differences in substance use by sexual orientation and gender among Jewish young adults in Israel. Isr J Health Policy Res.

[31] Hser Y-I, Huang D, Teruya C, Anglin MD (2003). Gender comparisons of drug abuse treatment outcomes and predictors. Drug Alcohol Depend.

